# Preventing Pluripotent Cell Teratoma in Regenerative Medicine Applied to Hematology Disorders

**DOI:** 10.5966/sctm.2016-0201

**Published:** 2016-09-07

**Authors:** Aurelie Bedel, François Beliveau, Isabelle Lamrissi‐Garcia, Benoit Rousseau, Isabelle Moranvillier, Benoit Rucheton, Veronique Guyonnet‐Dupérat, Bruno Cardinaud, Hubert de Verneuil, François Moreau‐Gaudry, Sandrine Dabernat

**Affiliations:** ^1^ Université de Bordeaux, Bordeaux, France; ^2^ INSERM U1035, Bordeaux, France; ^3^ Bordeaux University Hospital, Bordeaux, France; ^4^ Plateforme de Vectorologie, Université de Bordeaux, Bordeaux, France

**Keywords:** Induced pluripotent stem cells, Teratoma, Hematology, Hematopoietic stem cell, Survivin inhibitor, Suicide gene, iCaspase‐9, Thymidine kinase, Regenerative medicine, Safety

## Abstract

Iatrogenic tumorigenesis is a major limitation for the use of human induced pluripotent stem cells (hiPSCs) in hematology. The teratoma risk comes from the persistence of hiPSCs in differentiated cell populations. Our goal was to evaluate the best system to purge residual hiPSCs before graft without compromising hematopoietic repopulation capability. Teratoma risk after systemic injection of hiPSCs expressing the reporter gene luciferase was assessed for the first time. Teratoma formation in immune‐deficient mice was tracked by in vivo bioimaging. We observed that systemic injection of hiPSCs produced multisite teratoma as soon as 5 weeks after injection. To eliminate hiPSCs before grafting, we tested the embryonic‐specific expression of suicide genes under the control of the pmiR‐302/367 promoter. This promoter was highly active in hiPSCs but not in differentiated cells. The gene/prodrug inducible Caspase‐9 (iCaspase‐9)/AP20187 was more efficient and rapid than thymidine kinase/ganciclovir, fully specific, and without bystander effect. We observed that iCaspase‐9‐expressing hiPSCs died in a dose‐dependent manner with AP20187, without reaching full eradication in vitro. Unexpectedly, nonspecific toxicity of AP20187 on iCaspase‐9‐negative hiPSCs and on CD34^+^ cells was evidenced in vitro. This toxic effect strongly impaired CD34^+^‐derived human hematopoiesis in adoptive transfers. Survivin inhibition is an alternative to the suicide gene approach because hiPSCs fully rely on survivin for survival. Survivin inhibitor YM155 was more efficient than AP20187/iCaspase‐9 for killing hiPSCs, without toxicity on CD34^+^ cells, in vitro and in adoptive transfers. hiPSC purge by survivin inhibitor fully eradicated teratoma formation in immune‐deficient mice. This will be useful to improve the safety management for hiPSC‐based medicine. Stem Cells Translational Medicine *2017;6:382–393*

**Significance Statement**

Teratoma formation is a major concern limiting induced pluripotent stem cells‐based regenerative medicine in hematology. In this study, it was demonstrated that human induced pluripotent stem cells (hiPSCs) are highly teratogenic when injected in the systemic circulation. Two methods to eliminate hiPSCs before engraftment were compared. The first method was based on the embryonic‐specific expression of suicide genes and the second one used a survivin inhibitor. Results suggested that suicide gene‐related prodrugs showed toxicity compromising hematopoietic stem cell (HSC) engraftment in immune‐deficient mice. By contrast, survivin inhibitor was not toxic to HSCs and was more efficient at killing hiPSCs. These data may be useful to improve the safety management for hiPSC‐based medicine in hematology.

## Introduction

The major discovery that ectopic expression of specific transcription factors can induce the reprogramming of human somatic cells into induced pluripotent stem cells (iPSCs) has revolutionized the fields of gene therapy, regenerative medicine and disease modeling 1, 2). The use of these cells can bypass ethical concerns (by contrast to embryonic stem cells) and problematic allogeneic immune rejection. Hence, human iPSCs (hiPSCs) represent an ideal source to produce patient‐specific biological material. For example, in genetic hematologic disorders, we and others have developed potent protocols for in vitro hematopoietic differentiation of stem cells after they have been genetically corrected 3, 4, 5. However, several aspects hinder the progression of iPSCs into clinic. In particular, for hematopoietic applications, there is no current method of differentiation yielding a 100% pure population from a pluripotent donor source with regard with hematopoietic differentiation 4. Therefore, a population of differentiated cells is always contaminated by residual undifferentiated iPSCs. This becomes a critical hurdle to the application of iPSCs in clinical protocols because of their iatrogenic teratogenesis potential 6.

To reduce the risk of teratomas after engraftment of differentiated cells derived from iPSC, three strategies have predominantly been tested. The first one aims at selecting the differentiated by positive cell sorting or at withdrawing pluripotent cells by flow cytometry on the basis of pluripotent specific cell surface antigen. For example, complex protocols have been achieved to separate individually contaminating undifferentiated iPSCs from somatic cells by depletion of SSEA4^+^, TRA‐1‐60^+^ cells. However, with flow cytometry, the results were greatly affected by gating and only cells expressing the specific markers can be identified and this procedure could affect hematopoietic stem cell viability 7. This approach is time consuming and expensive 8. Alternatively, selection markers specific to differentiated cells were used positively select the right differentiated population 9. The second way to deplete a differentiated cell population from residual iPSCs is to use in vitro‐engineered stem cells armed with a suicide gene, whose activation can be triggered before transplantation to eliminate any residual nondifferentiated cells or after transplantation if teratomas develop. Ablation of teratomas derived from mouse embryonic stem (mES) cells transplanted in myocardium has been achieved by the suicide combination of thymidine kinase (TK) and ganciclovir (GCV) 10. The same system has been successfully used in vitro to eliminate undifferentiated human embryonic stem (hES) cells after neuron‐like differentiation, because the TK gene was under the control of the *OCT‐4* promoter 11. However, there was no further in vivo validation in terms of teratoma formation after suicide gene induction in the remaining transplant. Primate iPSCs were killed by inducible caspase‐9 (iCaspase‐9) or by the 5‐fluorocytosine/yeast cytosine deaminase combination in vitro. The second system was more efficient; however, it needed extended in vitro treatment to be fully efficient and was not suitable for the short in vitro holding time needed for fully competent hematological stem cells. It was further tested in vivo before and after teratoma formation and in human iPSCs by iCaspase‐9 12. Importantly, the introduction of suicide genes did not alter the pluripotency of the iPSCs and controlled the teratoma‐initiating iPSCs and their derivative in vivo, in testis injections. However, these suicide gene/prodrug couples were not tested in conditions mimicking hematopoietic cell therapy protocol (i.e., testing the efficiency of the prodrug/suicide gene couples to purge residual teratoma inducing iPSCs from differentiated cells). The third approach to eliminate contaminating residual iPSCs is to use diverse chemicals killing specifically pluripotent cells but sparing differentiated cells. Most of the compounds target apoptosis (reviewed in Malecki 13). One of them, YM155, an analog of quercetin, a chemical survivin inhibitor, seems particularly relevant to residual iPSCs elimination 14.

Application of gene and cell therapy in hematology is of particular interest because bone marrow transplantation has been widely developed and used. For the assessment of biodistribution potential and safety of hiPSCs, it is essential to administer the cells by the exact way that will be used in clinical applications. Transplantations aimed to correct hematologic disorders are carried out in systemic circulation, and no data have been published on the fate of embryonic stem cells or iPSCs transplanted intravenously.

In the present study, the teratogenic potential of monocellular suspensions containing high numbers of hiPSCs injected intravenously using bioluminescence determined spatial‐temporal whole body tumor distribution. We compared the efficiency of iPSC purge in vitro, before transplantation, by embryonic‐specific suicide gene expression or by survinin inhibition. In hematological applications, hematopoietic stem cells (HSCs) are the therapeutic cells. Importantly, we tested the toxicity of the suicide gene prodrugs toward human CD34^+^ cells, containing HSCs in vitro and the impact of purge treatment on adoptive transfer efficiency in immune‐compromised mice. Moreover, YM155 was not found to be cytotoxic on differentiated cells such as neurons 14, but it kills pluripotent stem cells. It could also be deleterious for other stem cells, such as HSCs. It was prerequisite to address this point in a purge strategy for elimination of residual iPSCs in HSC populations obtained from iPSCs.

## Materials and Methods

### Human Samples, Animals, Human Cells, and Pancreatic Cell Lines

The 8‐ to 12 week‐old NOD/Shi‐SCID IL2Rγ null (NSG) mice were produced and housed at the University of Bordeaux animal facility A2, according to the rules and regulations of the Institutional Animal Care and Use Committee (agreement no. A33063916). Human cord blood samples were used according to approval by the local institutional review board of Maison de Santé de Bagatelle (Talence, France). The study was approved by the ethics committee Comité Consultatif de Protection des Personnes dans la Recherche Biomédicale de Bordeaux at the university hospital.

Pancreatic cell lines origins have been described earlier 15. hiPSCs were obtained and cultured in embryonic stem (ES) medium as described previously 3. Human embryonic kidney (HEK) 293T cells were obtained from ATCC (Molsheim, France, https://www.atcc.org).

### CD34^+^ Cell Isolation

After Ficoll separation, CD34^+^ cells from cord blood were isolated with EasySep human CD34 selection kit (STEMCELL Technologies, Vancouver, Canada, https://www.stemcell.com) and cultured in StemSpan medium (STEMCELL Technologies), supplemented with cytokines.

### Flow Cytometry and Microscopy

Cells were washed with phosphate‐buffered saline (PBS)‐human serum albumin 1% and stained with a phycoerythrin‐conjugated anti‐CD34 (clone 4H11; eBioscience, San Diego, CA, http://www.ebioscience.com), brilliant violet‐conjugated anti‐human CD45 (BioLegend San Diego, CA, http://www.biolegend.com). Cells were analyzed on a flow cytometer (Canto II flow cytometer; BD Biosciences, San Jose, CA, www.bdbiosciences.com).

Cells were observed with an inverted Nikon fluorescence microscope (Eclipse Ti Nikon, Champigny sur Marne, France). Pictures were taken with the NIS‐Elements Nikon software (Nikon, Tokyo, Japan, http://www.nikon.fr).

### Human Cord Blood CD34^+^ Adoptive Transfers

Groups of 5 mice were injected with 20 mg/kg of busulfan (Busilvex; Pierre Fabre, Castres, France, http://www.pierre‐fabre.com) 2 days before and the day before hematopoietic cell injections. One hundred µl containing 10^5^ CD34^+^ cells pretreated or not pretreated with AP20187 diluted in ethanol (TaKaRa Clontech, Beijing, China, http://www.clontech.com) or YM155 diluted in dimethyl sulfoxide (Selleck Chemicals, Houston, TX, http://www.selleckchem.com) were injected in the retro‐orbital sinus of anesthetized mice. Twelve weeks after injections, mice were sacrificed and both femurs were collected to perform bone marrow isolation. Briefly, cells were flushed from the marrows, counted using a hemocytometer and stained with anti‐hCD45 antibody to determine percentages of human hematopoietic chimerism within murine cells.

### Human iPSCs’ Xenograft and Bioluminescence Imaging

Groups of four to five mice were anesthetized with isoflurane. 2 × 10^5^ or 2 × 10^6^ iPSCs in 100 μl ES medium with ROCK inhibitor (Y27632; Sigma‐Aldrich, St. Louis, MO, https://www.sigmaaldrich.com) were injected into the retro‐orbital sinus. Teratoma formation was monitored after 2 weeksand then every week until the experiment was stopped by sacrificing the mice. Luciferase expression was performed as follows: 150 mg/kg of d‐luciferin (Interchim, Montluçon, France, http://www.interchim.com) was injected intraperitonally. After 10 minutes, mice were anesthetized by isoflurane and placed into a photon bioimager (Biospace Laboratory, Paris, France, http://www.biospacelab.com) for approximately 20 minutes to acquire luminescence images. Bioluminescence detection in cells in 96‐well plates was performed after d‐luciferin was added directly in the wells (3 mg/l). Ex vivo organ imaging was carried out after the organs were incubated with d‐luciferin (3 mg/l) in PBS for 5–10 minutes at room temperature.

### Histology

Teratomas were fixed in 10% neutral‐buffered formalin, embedded in paraffin, and processed by routine histology procedures. Sections were stained with anti‐α1fetoprotein (DAKO, Les Ulis, France, http://www.dako.com), antivimentin (Sigma‐Aldrich), and anticytokeratin 5/8 (Cell Signaling Technology, Saint‐Quentin en Yvelines, France, https://www.cellsignal.com).

### Vector Construction, Production, and Transduction of Cells, and Copy Number Determination by Quantitative Polymerase Chain Reaction

The pmir(2kb)‐iCaspase9‐P2A‐tdTom vector was constructed by replacing *PGK* promoter with human *pmiR302/367* 2kb promoter (pmiR2kb‐tdTomato) in modified pRRLSIN.cPPT.PGK‐GFP.WPRE plasmid (gift from Didier Trono, Addgene plasmid no. 12252, https://www.addgene.org) expressing tdTomato in place of green fluorescent protein (GFP) (previously described by Lafitte et al. 16). pmiR302/367 2kb promoter (chr4:112648657‐112650715) was amplified from genomic DNA. Primers for amplification can be provided upon request. The iCaspase9‐P2A fragment was inserted in pmiR2kb‐tdTomato by Gibson Assembly (New England BioLabs France, Evry, France, http://www.neb‐online.fr). Briefly, pmiR2kb‐tdTomato plasmid was linearized between pmiR2kb and tdTomato by digesting with AgeI. Two blocks were inserted at the same time between pmiR2kb and tdTomato, one with the coding sequence of iCaspase‐9 amplified from pSH1/*S*‐Fvls‐p30Casp9‐E (gift from David Spencer, Addgene plasmid no. 15272)^2^ with homology arms for pmiR2kb in 5′ and P2A peptide in 3′ (pmiR302/367‐iCaspase‐9 FWD 5′‐CGAATCTTTGGGAACTAGTTCAGGA**ATGCTCGAGGGAGTGCAGGTG**‐3′ and pmiR302/367‐iCaspase‐9 P2A REV 5′‐GGCTGAAGTTAGTAGCTCCGCTTCC**GTCGAGTGCGTAGTCTGGTAC**‐3′); the second ordered as dsDNA (gBlocks Gene Fragments; Integrated DNA Technologies, Coralville, IA, http://www.idtdna.com) coding for 2A peptide derived from porcine teschovirus‐1 (P2A) with Gly‐Ser‐Gly added to the end of the 2A sequences to improve cleavage efficiency 17 and having homology arms for iCaspase‐9 in 5′ and tdTomato in 3′.

### Real‐time Quantitative Polymerase Chain Reaction Detection of “Vector” Provirus by Relative Quantification Methods

DNA was isolated with the NucleoSpin Tissue kit (Macherey‐Nagel, Hoerdt, France, www.mn‐net.com) from 1 × 10^6^ cells, and real‐time polymerase chain reaction (PCR) was performed on 100 ng of DNA with the use of TaqMan technology (Thermo Fisher, Waltham, MA, http://www.thermofisher.com). DNA from nontransduced cells was isolated and amplified at the same time for demonstrating the absence of contamination. Samples were heated to 95°C for 3 minutes, and then 40 cycles of PCR were performed for 5 seconds at 95°C and for 30 seconds at 60°C to obtain Lenti amplimers with the following primers and probe: Lentiviral.F GGAGCTAGAACGATTCGCAGTTA; Lentiviral.R: GGTTGTAGCTGTCCCAGTATTTGTC; Lentiviral probe: ACAGCCTTCTGATGTTTCTAACAGGCCAGG. RNaseP was used for normalization (TaqMan RNase P Control Reagents Kit; Thermo Fisher). DNA from two different cell clones (human 293T and K562 cells), containing a single integrated copy of the provirus, was used as a standard.

### Statistical Analysis

Results are expressed as mean ± SD. All statistical tests were performed with unpaired, bilateral Student's *t* tests.

## Results

### Monocellular Suspensions of hiPSCs Are Highly Teratogenic When Injected in the Systemic Circulation

hiPSCs can undergo hematopoietic differentiation in vitro to produce HSCs, hematopoietic progenitors, and differentiated cells. However, current protocols do not yield 100% efficiency and residual hiPSCs remain in differentiated cell populations 18. When differentiated cells are to be transplanted by intravenous injections, these pluripotent cells carry the risk of teratoma formation. Although teratoma‐forming capabilities of pluripotent cells has been previously assessed by grafting cells in solids organs such as testis, muscle or in subcutaneous injections, systemic delivery was never carried out.

Site of teratoma development after i.v. injections of iPSCs cannot be predicted. Thus, we needed to produce iPSCs with a reporter gene allowing for the detection of very few cells using bioimaging. Cells were transduced with a lentivector carrying the luciferase gene under the control of the strong ubiquitous promoter pMND so that luciferase is easily detectable in all the differentiated cells in teratomas. Luciferase coding region was followed by puromycin resistance gene and transduced cells were selected by puromycin treatment (supplemental online Fig. 1A). Sensitivity for luciferase detection in undifferentiated iPSCs was performed in a 96‐well plate seeded with known numbers of cells (supplemental online Fig. 1B). We found that as little as 300 iPS‐Luc cells were sufficient to generate luminescence signal at least twofold more intense than the background signal and readily observable. Moreover, to evaluate luciferase detectable signals from cells present in bones, a solid mineralized tissue, we injected 1000 cells in a femur and imaged the bone in vitro (supplemental online Fig. 1Ca), or we injected 10,000 cells and imaged the live mouse immediately after injection (supplemental online Fig. 1Ca–1Cb). Luciferase activity was visualized by bioluminescence using the bioimager. Second, we compared the early distribution of i.v. injected cells in the tail vein or in the retro‐orbital sinus. Fifteen minutes after injections, cells were massively present in the lungs, and only a small proportion stayed at the injection site (supplemental online Fig. 1Cc).

**Figure 1:**
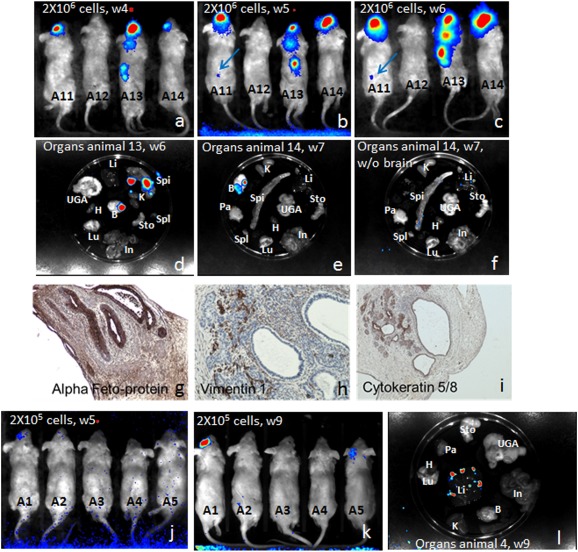
Monocellular suspensions of human induced pluripotent stem cells (hiPSCs) are highly teratogenic when injected in the systemic circulation. Luciferase‐expressing hiPSCs were injected i.v. in the retro‐orbital sinus of recipient NSG mice (**A, B, C:** 2 × 10^6^ cells or **J, K, L:** 2 × 10^5^ cells) and imaging was performed on live animals at several time points after injection (**A:** 4 weeks; **B, J:** 5 weeks; **C:** 6 weeks; and **K:** 9 weeks). For some animals, organs were collected and imaged directly after 5–10 minutes in luciferin‐containing phosphate‐buffered saline incubation (**D, E, F,** and **L**). Brain teratoma from animal 13 was fixed and sectioned and immunohistochemistry showing the presence of several tissues was performed with anti‐α‐fetoprotein (**G:** endoderm; original magnification ×200), antivimentin‐1 (**H:** connective tissues but epithelium, original magnification ×200), and anticytokeratin 5/8 (**I:** epithelial structures; original magnification ×100). Abbreviations: B, brain; H, heart; In, intestine; K: kidneys; Li, liver; Lu, lungs; Pa: pancreas; Spi: spine; Spl: spleen; Sto: stomach; UGA: urogenital tract, w, week.

To test the teratogenic potential of iPSCs delivered intravenously, it was necessary to inject monocellular suspensions to avoid embolic cell clusters. In our hands, injecting approximately 10^6^ iPSCs under the skin of NSG mice led to successful teratoma formation 12 to 16 weeks after injection 3. Two groups of 4 mice were injected with either 2 × 10^6^ cells per injection or 2 × 10^5^ cells per injection i.v. in the retro‐orbital sinus. Four weeks after the injections mice were analyzed for teratoma presence by i.v. luciferin injections and bioimaging. Three of 4 mice injected with 2 × 10^6^ cells showed luciferase signals (Fig. 1A–1C). All positive mice had detectable iPSCs‐derived cells at the site of injection, but very interestingly 1 animal (animal A13) had multiple engraftment sites, visible as soon as week 4 and growing from week 4 to week 6 as evidenced by enhanced signal intensity. Animal A11 showed only one engraftment site at week 4 (the injection site) but another site was visible from week 5 on (blue arrows). At the time of sacrifice, organs were analyzed separately for luciferase expression to determine the location of engraftment sites. Tumors were present in the spine, brain, kidney, and liver (Fig. 1D–1F). Histologic and immune‐histochemical examinations of the teratomas evidenced cells from all three germ layers (Fig. 1G–1I). Therefore, two thirds of the positive mice displayed engraftment distant from the i.v. injection site. When 2 × 10^5^ cells were injected i.v., at week 6, only 1 mouse showed cell engraftment at the injection site (Fig. 1J), and 2 at week 9 (Fig. 1K). Interestingly, animal A4 died during the anesthetic procedure at week 9, but imaging of its internal organs showed a weak LUC signal in the liver (Fig. 1L).

Thus, 2 × 10^5^ iPSCs are sufficient to induce teratoma formation when injected i.v. Tumor development occurred in multiple places with a trend to locate in the nervous system, despite an early accumulation in the lungs. These results clearly show that residual iPSCs carried a high teratoma risk when injected i.v.

### The Ganciclovir/Thymidine Kinase Couple Presents a Bystander Effect While AP20187/iCaspase‐9 Does not in HEK293T Cells

**Figure 2:**
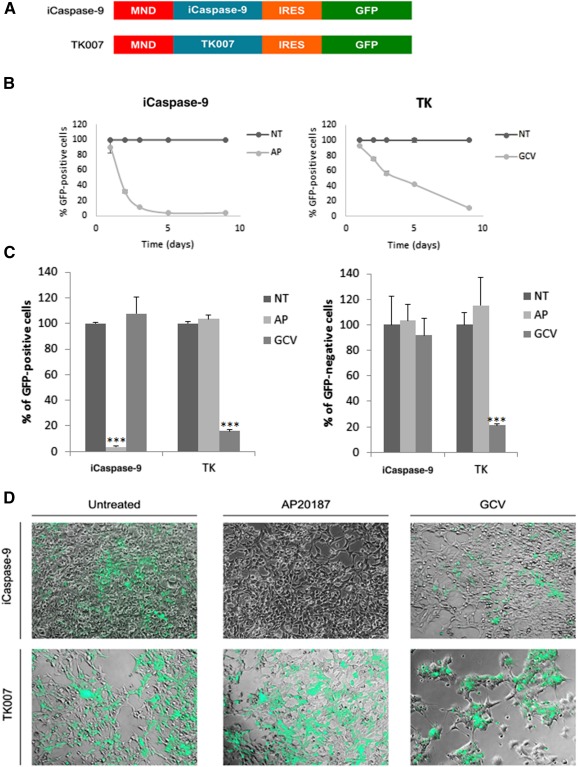
The ganciclovir/thymidine kinase couple presents a bystander effect, whereas AP20187/iCaspase‐9 does not in HEK293T cells. **(A):** Either the iCaspase‐9 suicide gene coding sequence or the codon‐optimized TK007 coding sequence were placed under the control of the strong ubiquitous pMND (myeloproliferative sarcoma virus enhancer, negative control region deleted) promoter. IRES allowed the production of bicistronic mRNAs for GFP expression together with the suicide genes. **(B):** 293T HEK cells were transduced and treated for several days with prodrugs AP20187 (1 nM) or GCV (1 µM) or were treated with vehicle. Mean values ± SD of the percentages of GFP‐positive remaining cells are plotted against time in days. **(C):** Prodrug specificity was tested by treating for 9 days cells transduced with either suicide genes with AP20187 or GCV. Mean values ± SD of the percentages of GFP‐positive remaining cells (left) or GFP‐negative cells are represented as histogram bars. ∗∗∗, *p* < .01 with Student's *t* tests against the NT condition. **(D):** Representative photos of cells transduced with iCaspase‐9 (top) or with TK007 (bottom) lentivectors, after 9 days of treatment with the indicated prodrugs. Original magnification ×200. Transduced cells are green. Abbreviations: AP, AP20187; GCV, ganciclovir; GFP, green fluorescent protein; iCaspase‐9, inducible Caspase‐9; NT, treated with vehicle; TK, thymidine kinase.

To choose the best suicide gene candidate for elimination of residual iPSCs before engraftment, we tested two suicide genes under a constitutive promoter in HEK293T cells. Transduced cells expressed the fluorescent GFP reporter gene (Fig. 2A). We established a time response by flow cytometry in vitro after treatment with the prodrugs, with previously determined minimal doses reaching maximal toxicity. Importantly, iCaspase‐9 deadly action occurred much faster than TK007, a human codon optimized version of the TK gene 19 and left no live cells at day 5, whereas 50% cells are still alive with GCV treatment (Fig. 2B). There was no mortality when iCaspase‐9 expressing cells were treated with GCV and when TK007‐expressing cells were treated with AP20187, at the efficient doses, showing the specificity of the suicide gene/prodrug couples (Fig. 2C, left). Noticeably, however, strong bystander effect was observed with TK007 expressing cells but not with iCaspase‐9 (Fig. 2C, right, and Fig. 2E), because a high proportion of nontransduced GFP‐negative cells died in the presence of ganciclovir and TK‐expressing cells.

These results suggest that it is preferable to use iCaspase‐9 suicide gene to eliminate residual iPSCs in a population of differentiated cells before grafting to avoid the destruction of the graft that might occur by the bystander effect observed with TK007. Moreover, iCaspase‐9 action is much faster that TK007, which is mandatory to keep high quality of the ready‐to‐graft HSCs.

### The Promoter Region of pmiR‐302‐367 Is Specifically Active in Embryonic Stem Cells but Not in HSCs

**Figure 3:**
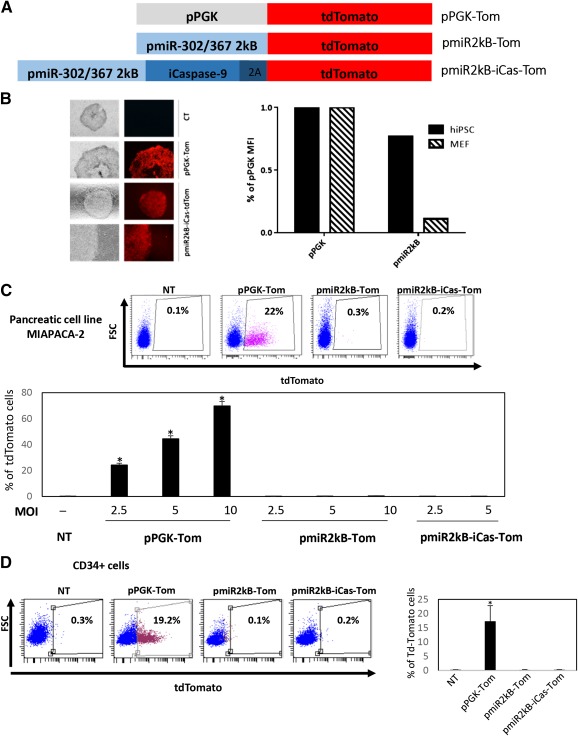
The promoter region of pmiR‐302‐367 is specifically active in embryonic stem cells, but not in hematopoietic stem cells or pancreatic tumor cells. **(A):** iPSCs were transduced with lentivectors carrying the tdTomato reporter gene under the control of the pPGK ubiquitous gene, or the pmiR302‐367 embryonic‐specific promoter. The third construct carries the iCaspase‐9 suicide gene and the 2A sequence upstream the reporter gene. **(B):** Left panel: iPSCs were transduced with pmiR2kB‐iCas‐Tom or the pPGK‐Tom lentivectors. Transduced cells were sorted by flow cytometry, and fluorescence was observed by microscopy using the same gain (CT: untransduced cells). Right panel: MFIs were measured by flow cytometry and expressed as a ratio of the pPGK MFI. **(C):** pmiR2kB‐Tom activity was tested in pancreatic cells at increasing multiplicities of infection (MOIs), compared with pPGK. Top: Flow cytometry dot plots with % tdTomato‐positive cells. Bottom: Bars represent mean ± SD % of tdTomato‐positive cells with increasing MOIs. **(D):** pmiR2kB‐Tom activity was tested in hCD34^+^ cells, compared with pPGK. Left: Flow cytometry dot plots with % tdTomato‐positive cells. Right: Bars represent mean ± SD % of tdTomato‐positive cells. ∗, *p* < .05. Abbreviations: hiPSC, human induced pluripotent stem cells; iCaspase‐9, inducible Caspase‐9; iPSCs, induced pluripotent stem cells; MFIs, mean fluorescent intensities; NT, nontransduced cells; pPGK, phosphoglycerate kinase.

The suicide gene expression‐driven iPSCs purge in a differentiated cell population necessitates the use of a strong embryonic‐specific promoter. Pluripotent stem cell‐specific expression of a suicide gene under the control of an embryonic promoter is supposed to specifically kill iPSCs while sparing differentiated cells, when appropriate prodrug of the suicide gene is added. We cloned 2kb a promoter of the embryonic‐specific miRNA cluster 302‐367 (pmiR‐302/367 20). We developed two lentivectors based on pmiR302/367: one with iCaspase‐9 and tdTomato reporter gene (pmiR2kB‐iCas‐Tom) and one with the reporter gene only (pmiR2kB‐Tom, Fig. 3A). pmiR‐302/367 activity was compared with the ubiquitous expression lentivector pPGK‐Tom. When transduced in iPSCs, both vectors were similarly active in iPSCs as evidenced by tdTomato fluorescence (Fig. 3B). Strength of signal was comparable between phosphoglycerate kinase (pPGK) and pmiR2kB. iPSCs but not human fibroblasts transduced with pmiR2kB‐Tom expressed tdTomato (supplemental online Fig. 2). When the lentivectors were used to transduce differentiated cells such as human pancreatic cell lines (MiaPaCa‐2 and Capan‐2 cells) or mouse embryonic fibroblasts, tdTomato was detected only when the reporter gene was under the control of pPGK (Fig. 3B–3C, data not shown for Capan‐2 cells), even with high multiplicities of infection (MOIs). Importantly, as we plan to differentiate cells in the hematopoietic lineage, we looked at the activity of the lentivectors in CD34^+^ cells isolated from cord blood. Expectedly, pmiR‐302/367‐driven expression of tdTomato was absent with two different lentivectors (pmiR2kB‐icas‐Tom and pmiR2kB‐Tom) whereas pPGK was active (Fig. 3D). To further attest that lack of tdTomato expression was due to inactivity of pmiR‐302/367 in CD34^+^ cells, genomic DNA of transduced cells was extracted and examined by real‐time quantitative polymerase chain reaction and copy numbers were quantified. We found that although no tdTomato expression was detected by flow cytometry in pmiR‐302/367‐vector‐transduced cells, an average of 2 DNA copies of the lentivirus were present in the cells (ubiquitous or embryonic specific lentivectors, data not shown). Thus, the pmiR302‐367 promoter fragment was active in iPSCs and inactive in differentiated cells, in particular in CD34^+^ cells.

### iCaspase‐9 Dimerization Drug AP20187 Presents an Unspecific Toxic Effect on iCaspase‐Negative iPSCs or CD34^+^Cells

**Figure 4:**
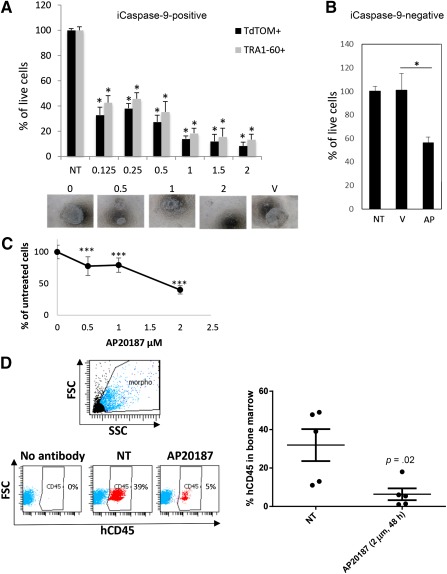
iCaspase‐9 dimerization drug AP20187 presents an unspecific toxic effect on iCaspase‐negative iPSCs or CD34^+^ cells. **(A):** Transduced iPSCs were treated with increasing doses of AP20187 (µM) for 48 hours. Toxicity was assessed by counting by flow cytometry tdTomato‐positive or Tra1‐60‐positive cells. **(B):** Percentage of live cells was determined in the iCaspase‐9‐negative cell population (expressed as mean ± SD % of nontransduced cells). **(C):** hCD34^+^ cells were treated with increasing doses of AP20187. Results are expressed as mean ± SD percentages of untreated cells. ***p < .01 as compared by a Student's *t* test with the untreated condition. **(D):** hCD34^+^ cells were treated with a single dose of AP20187 (2µM, 48 hours) toxic for iPSCs and grafted by i.v. injections in NSG mice. Eight weeks after the injections, bone marrow cells obtained from the femurs were analyzed by flow cytometry with anti‐hCD45 pan hematologic marker. Left: Representative dot plots are presented with % of hCD45‐positive cells. Right: Mean ± SD of hCD45‐positive cells for each condition. ∗, *p* < .05, *n* = 5 mice in each group. Abbreviations: iCaspase‐9, inducible Caspase‐9; iPSCs, induced pluripotent stem cells; NT, treated with vehicle; V, vehicle.

To establish in vitro toxicity of the iCaspase‐9 in iPSCs, cells transduced with pmiR2kB‐icas‐Tom were incubated with increasing doses of the inducer AP20187. To distinguish pluripotent from differentiated cells during analysis (in particular the feeders), cells were stained with the Tra1‐60 pluripotent cell marker. We found that approximately 90% of the tdTomato‐positive cells were Tra‐1‐60 positive. Reversely, approximately 75% of the Tra‐1‐60‐positive cells were tdTomato positive (not shown). Thus the majority of the analyzed cells were double positive. Percentages of tdTomato^+^ and Tra1‐60^+^ cells decreased in the same way in a dose‐dependent manner (Fig. 4A), with maximum but not full cell toxicity obtained at 2 µM. Unexpectedly, iCaspase‐9 negative cells were also killed by AP20187 (Fig. 4B). This prompted us to test AP20187 toxicity on CD34^+^ cells, because residual iPSCs elimination will be carried out in the differentiated cell population before graft. First, freshly isolated CD34^+^ cells from fetal cord blood were exposed to increasing doses of AP20187. A dose‐dependent toxicity was observed with a very high impact on cell survival at 2 µM, which is the efficient dose on iPSCs, because more than half the cells died within 48 hours of exposure (Fig. 4C). To further test the impact of AP20187 pretreatments on hematopoietic stem cell compartment within the CD34^+^ cells, adoptive transfers were carried out in conditioned immune compromised NSG mice. As shown in Figure 4D, cells exposed to AP20187 had a lower engraftment capacity because less than 5% of human CD45^+^ cells were found in peripheral blood (not shown) and bone marrow of mice grafted with AP20187 pretreated cells (vs. approximately 33% when cells were not pretreated).

Because iCaspase‐9 dimerization agent is toxic for CD34^+^ cells, using AP20187/iCaspase‐9 drug/suicide gene combination is not recommended for iPSCs purge in hematology applications.

### The Survivin Inhibitor YM155 Is Very Efficient in Killing iPSCs and Spares HSCs

**Figure 5:**
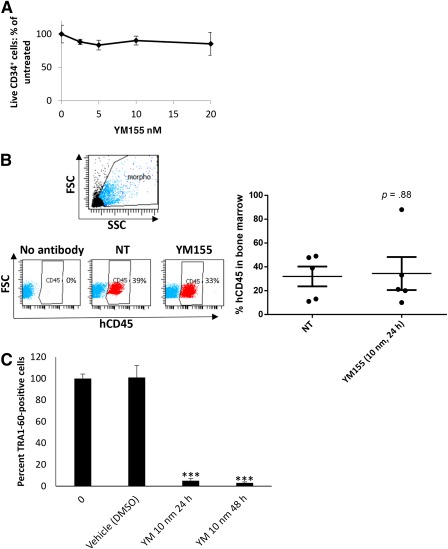
The survivin inhibitor YM155 is very efficient in killing iPSCs and spares hematopoietic stem cells. **(A):** hCD34^+^ cells from cord blood were cultured in the presence of increasing doses of YM155 (24 hours). Cell viability was evaluated by direct counting with Turque Blue vital stain. Results are expressed as mean ± SD percentages of untreated cells. **(B):** hCD34^+^ cells were treated with a single dose of YM155 (10 nM) toxic for iPSCs and grafted by i.v. injections in NSG mice. Eight weeks after the injections, bone marrow cells obtained from the femurs were analyzed by flow cytometry with anti‐hCD45 pan hematologic marker. Left: Representative dot plots are presented with % of hCD45‐positive cells. Right: Mean ± SD of hCD45‐positive cells for each condition, *n* = 5 mice in each group. **(C):** hiPSCs were treated with a single dose (10 nM) of YM155 for 24 hours or 48 hours. Cells were stained with anti‐TRA1‐60 and % of positive cells were determined by flow cytometry. ∗, *p* < .05; ∗∗∗, *p* < .005. Abbreviations: iPSCs, induced pluripotent stem cells; NT, treated with vehicle.

A gene expression profiling identified the antiapoptotic factors survivin and Bcl10 preferentially expressed in hPSC, and their specific inhibition induced selective apoptosis of undifferentiated hPSCs 14. To test survivin inhibition as an alternative way to purge residual iPSCs in hematology applications, YM155 was first incubated at increasing doses with CD34^+^ cells. Survivin inhibitor did not affect the viable CD34^+^ cell numbers, even at high concentrations (Fig. 5A). In addition, adoptive transfers showed no significant difference in human cell numbers in mice grafted with YM155 pretreated CD34^+^ cells as compared with non‐pre‐treated cells (Fig. 5B). Finally, a single active dose of 10nM (PNAS) for 24 hours or 48 hours efficiently killed Tra1‐60^+^ cells (Fig. 5C).

Thus, survivin inhibition was a much better approach than suicide gene strategy to purge iPSC‐derived HSCs before grafting because it is not toxic for survival or engraftment capacities of HSCs.

### Purge of iPSCs In Vitro, Before Systemic Transplantation Completely Prevents Teratoma Formation

**Figure 6:**
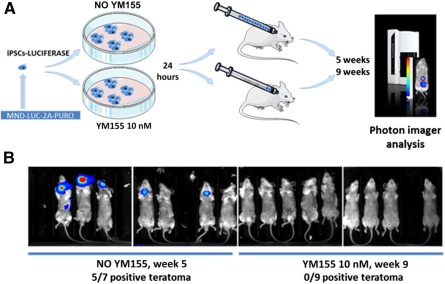
Purge of iPSCs in vitro, before systemic transplantation completely prevents teratoma formation. **(A):** Schematic representation of the experimental schedule for testing YM155 in preventing teratoma formation in vivo. Human induced pluripotent stem cells expressing luciferase were plated in equal numbers in sixteen 10‐mm dishes and cultured for 10 days, until colonies reached approximately 1 mm width, containing approximately 2 × 10^6^ cells. Nine plates were treated with YM155 (10 nM) for 24 hours, and 7 plates were not. Mice were injected i.v. with the whole content of monocellular suspensions from each plate, one plate per mouse. **(B):** Luciferase signal presence was assessed by bioimaging the mice 5 or 7 weeks after the injections. Abbreviation: iPSCs, induced pluripotent stem cells.

We demonstrated that YM155 was more potent to eliminate iPSCs while sparing human CD34^+^ cells in vitro and it did not affect CD34^+^ cell engraftment capabilities. A final validation step was to pretreat a high number of iPSCs sufficient to form teratomas after i.v. injection. Five weeks after injections, nontreated iPSCs developed teratomas in 5 of 7 animals, whereas no luciferase signal could be detected in the animals injected with YM155 pretreated iPSCs (more than 9 animals; Fig. 6). At week 9, these animals were still negative for luciferase signal (Fig. 6) and remained negative during a 40‐week follow‐up period.

Thus, YM155 was very efficient in preventing teratoma formation from iPSCs otherwise very potent in developing tumors in 5 weeks.

## Discussion

Numerous studies have been recently carried out on the use of pluripotent stem cells as a source of cells for regenerative medicine, in particular for hematologic disorders currently necessitating allografts. Although the risk of cancer in this therapeutic approach is acknowledged, because residual iPSCs can remain in the differentiated cell population, little attention has been paid to evaluate the biodistribution of undifferentiated cells after transplantation by systemic way. Studies have been aimed to determine threshold numbers of undifferentiated ES cells necessary for teratoma formation in xenogenic models of ES cell transplantations. Several injection routes have been tested, such as injecting mouse ES cells subcutaneously or under the kidney capsule 21. Intramyocardial injections of various numbers of hES cells compared with intramuscular injections yielded differences in both the occurrence and the rate of teratoma formation. Thus the route of administration can impact teratoma formation 22. The number of cells and the number and nature of coinjected cells can also affect greatly the teratogenic properties of PSCs. At least 100,000 cells were needed to observe detectable teratomas after 3–4 weeks postgraft, which persisted at least for 1 year. Only 10,000 hES cells were sufficient to achieve the same results in intramuscular transplantations 22. In the present study, we found that 2 × 10^5^ cells injected i.v. were sufficient to form teratomas detectable by bioimaging after 5 weeks. However, the rate of the number of teratomas and their size were impacted by cell numbers because injections of 10× more cells resulted in multisite bigger tumors. This is in accordance with the fact that 1/4,000 undifferentiated human ES cells (0.025%) mixed with nontumorigenic feeder fibroblasts were sufficient to induce teratoma formation in solid organ injections 23. In clinical application, approximately 200 × 10^6^ cells are needed for HSCs grafts in an adult. If HSCs are obtained from iPSCs and only 0.1% are residual iPSCs (i.e., 2 × 10^5^ cells), it would be sufficient to create teratomas in the absence of purge, as demonstrated by the present work. It would be interesting in future to graft lower numbers of undifferentiated hiPSCs to determine the minimum number of cells needed to form spontaneous teratomas when administrated i.v., but we showed that use of survivin inhibitor is probably sufficient to prevent teratoma risk.

Interestingly, it has been shown before that the sites of PSCs injection might vary according to heterogeneity of local factors, stroma, nutrients, and others. We observed differences in sizes of teratoma depending on the engraftment site, with bigger tumors when cells grew in nervous tissues (brain, spine) than in intra‐abdominal tissues, which could highlight a “natural” tropism preference of hiPSCs. This is in accordance with previous observations that environment of central nervous system, especially postischemic, might promote teratoma formation from undifferentiated ES cells 24.

Prodrug/suicide genes combinations have been proposed as a safety measure to eliminate risk of teratoma formation. In our focused application for hematology, we tested two suicide genes with expression driven by an embryonic promoter as active as the PGK ubiquitous promoter. When transduced at moderate MOIs, the TK007 showed a strong bystander effect in HEK293T cells that we considered as an inacceptable risk of destroying differentiated cells together with the unwanted residual iPSCs in a purge strategy. Moreover, ganciclovir was 100% toxic on CD34‐positive cord blood cells at 10 µM (data not shown). The dose needed to eliminate 100% of iPSCs in vitro >15 µM 25. Cheng et al. also found that ganciclovir treatment was needed at least for 5 days to kill a maximum number of cells. Such a long period of in vitro cell culture may compromise the quality and capacities of engraftment of HSCs. We selected iCaspase‐9 as a better suicide gene candidate because iCaspase‐9 did not exhibit any bystander effect, and cell toxicity occurred faster than with TK007. It was found earlier that iCaspase‐9 was not very efficient in killing primate iPSCs 12. However, the authors counted the percentage of annexin V‐positive cells which raised in time, but not to the level of the other tested suicide gene approach in their study. Here, we report that iCaspase‐9 was indeed toxic for hiPSCs, but complete eradication of cells was not achieved even at high concentrations (2 µM), similar to what was observed in Zhong et al. 12. This concentration is much higher than that needed to kill HEK293T cells (our study, 1 nM) or T cells 26. It is possible that pluripotent stem cells are more resistant to iCaspase9‐induced apoptosis than differentiated cells, likely because of the inhibitor of apoptosis protein survivin highly expressed in iPSCs 14. The biggest drawback we found was that the iCaspase‐9 dimerizing agent was highly toxic on iCaspase‐9 negative iPSCs and CD34^+^ cord blood cells, rendering this strategy inapplicable for withdrawal of residual undifferentiated cells after HSCs differentiation. This set of data highlights the necessity of testing prodrug toxicity on the differentiated cells before choosing any purge strategy with suicide genes.

The sensitivity of stem cells to different genotoxic drugs is increasingly explored for pharmacological purging of teratoma‐forming cells. Identification of pathways strictly needed for undifferentiated iPSCs survival led to the conclusion that survivin inhibitors could induce specific apoptosis of pluripotent cells but spared differentiated cells 14. We tested this approach in the present work and found that survivin inhibitor YM155 did spare CD34^+^ cord blood cells, did not affect their engraftment properties, and completely eradicated in vivo teratoma formation from i.v. injected hiPSCs. Interestingly, YM155 is a promising anticancer agent, with antitumor activity or sensitization to current therapies 27. Other drugs widely used in anticancer clinical practice, such as etoposide, which is an inhibitor of DNA topoisomerase II activity 28 or, more recently, purvalanol (dinaciclib for in vivo applications), which is a CDK1 inhibitor, could be alternatively used, or used in combination, to maximize residual pluripotent cell purge and further minimize in vivo posttransplant teratoma risk 29.

## Conclusion

Our work shows that iPSCs injected intravenously can form teratomas in various locations. Purging residual iPSCs with pharmacological inhibitors seems to be very promising for clinically relevant approaches in controlling posttransplantation teratoma risk. In contrast to prodrug/suicide gene, there is no insertion of an exogenous gene in the genome of the pluripotent cells, specific toxicity on residual iPSCs occurs very quickly, and treatment does not impair HSCs survival and functions.

## Author Contributions

A.B.: conception and design, collection, analysis, and interpretation of data, final approval of the manuscript; F.B.: conception and design, collection, analysis, and interpretation of data; I.L.G., I.M., and B. Rucheton, collection and assembly of data; B. Rousseau: design, collection and analysis of data; V.G.‐D. and B.C., provision of study material; H.d.V.: conception, financial support, final approval of the manuscript; F.M.‐G., conception and design, collection, analysis, and interpretation of data, financial support, final approval of the manuscript; S.D., conception and design, collection, analysis, and interpretation of data, manuscript writing.

## Disclosure of Potential Conflicts of Interest

The authors indicated no potential conflicts of interest.

## Supporting information

Supporting Information
