# Supplementary material for: Preventing Pluripotent Cell Teratoma in Regenerative Medicine Applied to Hematology Disorders
Source: Stem Cells Transl Med. 2016 Sep 7;6(2):382–93. doi: 10.5966/sctm.2016-0201 (PMC5442801; doi:10.5966/sctm.2016-0201)
Supplement: Supplementary file 1 — Supporting Information [file SCT3-6-382-s001.pdf]

Supplemental Figures - Moreau-Gaudry et al.

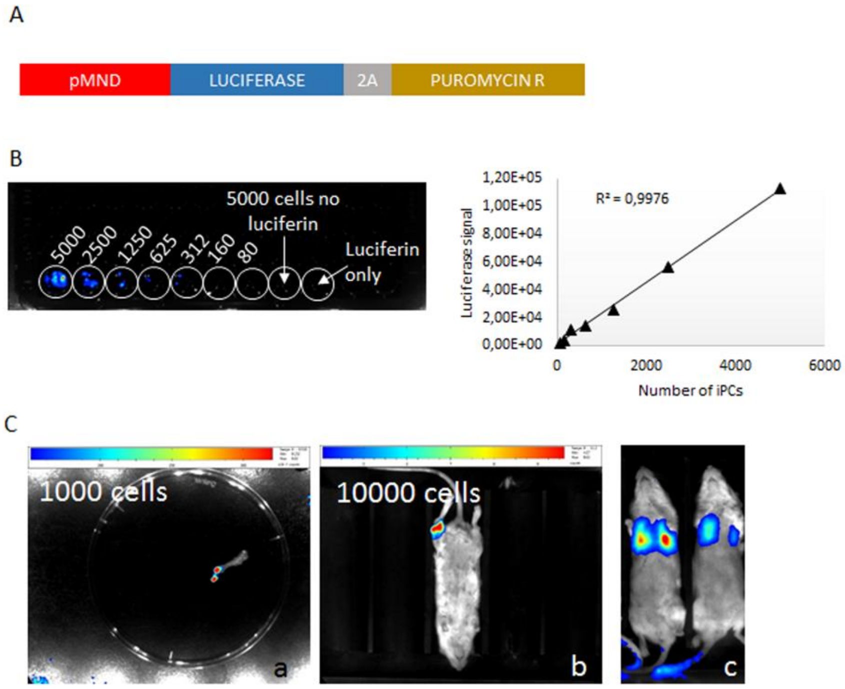

Figure S1: Sensitivity of luciferase signal detection *in vitro*, *in situ* and *in vivo*.

A) Induced pluripotent stem cells were transduced with a lentivector carrying the luciferase reporter gene and the puromycin resistance gene under the control of the ubiquitous viral promoter pMND. The 2A sequence encodes a self-cleaving peptide. B) Serial dilution of luciferase-positive iPSCs were plated in 96-well plates and bioluminescence was measured immediately after luciferin was added in the wells. Signals were quantified in each wells and were plotted according to cell numbers. A good linear regression was observed with a R^2=0.9976. C) a: 1000 iPSCs were injected in the femur head of a living mouse, then the bone was collected and imaged directly after 5 min incubation in luciferin-containing PBS. b: 10000 iPSCs were injected the the femur head of a living mouse and imaging was performed after IV injection of luciferin in the anesthetized living animal. c: luminescent cells were injected IV in the retro-orbital sinus (left) or in the tail vein (right). Live imaging of the animals was performed directly after they were injected IV with luciferin. Note that the signal is mostly located in the lungs, regardless of the injection site.

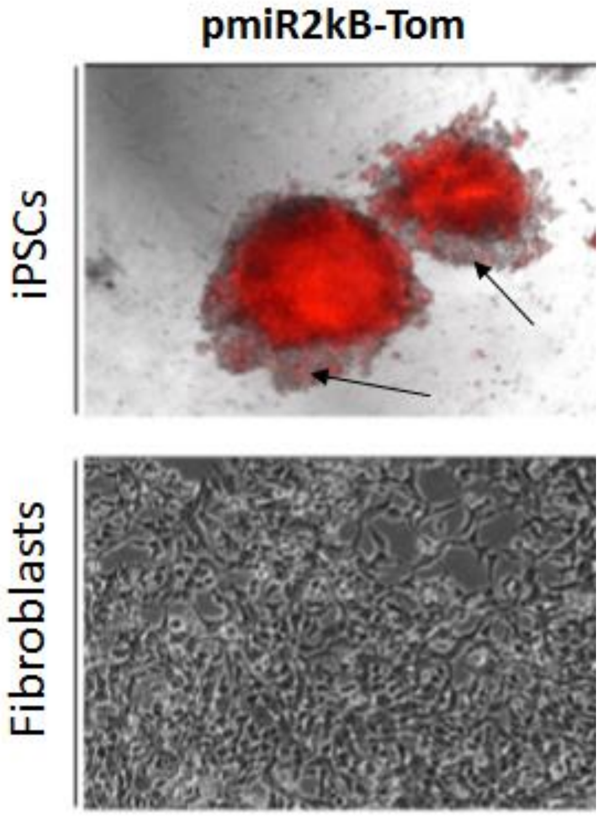

Figure S2: pmiR2kB-Tom is active in iPSCs.

iPSCs (top original magnification X 40) or fibroblasts (bottom original magnification X 100) were transduced with pmiR2kB-Tom. iPSCs were sorted by flow cytometry according to tdTomato expression. Transduced fibroblasts show no tdTomato expression while iPSCs are tdTomato-positive. Please note that when differentiating (clone periphery), cells lose red fluorescence suggesting extinction of pmiR2kB activity (arrows).
